# Botulinum-Toxin-Induced Muscle Fasciculations in Children with Cerebral Palsy: A Case-Series Study

**DOI:** 10.3390/brainsci15101048

**Published:** 2025-09-26

**Authors:** Giulia D’Amario, Giulia Brunozzi, Martina Procaccini, Marianna Villa, Chiara Monaco, Francesca Sini, Chiara Velli, Claudia Brogna, Domenico Marco Romeo

**Affiliations:** 1 Pediatric Neurology Unit, Fondazione Policlinico A. Gemelli, IRCCS, 00168 Rome, Italy; giulia.damario01@icatt.it (G.D.); giulia.brunozzi01@icatt.it (G.B.); martina.procaccini01@icatt.it (M.P.); marianna.villa@guest.policlinicogemelli.it (M.V.); chiara.monaco01@icatt.it (C.M.); francesca.sini@policlinicogemelli.it (F.S.); chiara.velli@guest.policlinicogemelli.it (C.V.); claudia.brogna@policlinicogemelli.it (C.B.); 2 Pediatric Neurology Unit, Università Cattolica del Sacro Cuore, 00168 Rome, Italy

**Keywords:** cerebral palsy, botulinum toxin, muscle fasciculations, case reports

## Abstract

Cerebral palsy (CP) is a group of permanent, but not immutable, disorders of movement and posture caused by a non-progressive lesion or an abnormality in the development of the immature brain. It is the leading cause of motor disability in childhood. The most common sign is muscle spasticity, which leads to the development of contractures and bone deformities. Among the treatments for spasticity, botulinum toxin injection has the strongest scientific evidence and is used from the age of 2 years. In this article, we report three pediatric patients with CP who developed muscle fasciculations after botulinum toxin injection, which were administered according to planned dosages and required clinical monitoring and stretching by a healthcare professional. We review the literature and analyze the possible physiopathological mechanisms; however no other similar clinical cases have been reported. In conclusion, we propose to include muscle fasciculations as a potential transient and non-harmful adverse effect of botulinum toxin infiltration, which remains the treatment of choice for muscle spasticity when combined with rehabilitation in cerebral palsy.

## 1. Introduction

Cerebral palsy (CP) affects approximately 2 to 3 per 1000 live births worldwide, making it the most common cause of motor disability in childhood [1]. It is defined as an early-onset, lifelong neurodevelopmental condition. It comprises a group of permanent, though not immutable, disorders of movement and posture resulting from non-progressive lesion or an abnormality in the development of the immature brain (maldevelopment attributed to dysplasia or injury to the fetal or infant brain) [2,3,4,5]. Although the primary brain lesion is non-progressive, the secondary musculoskeletal impairments are progressive [6]. The clinical manifestations include spasticity, dystonia, choreoathetosis, and/or ataxia, which may evolve with age. Beyond motor dysfunction, individuals with CP often present with additional primary and secondary impairments, including musculoskeletal deformities, chronic pain, epilepsy, and cognitive or sensory impairments, that significantly affect daily functioning and participation. Mortality is mainly related to severe forms of CP with profound motor impairment, feeding difficulties, and associated comorbidities, particularly respiratory complications.

The most common clinical sign is muscle spasticity, which leads to the development of contractures and bone deformities [7,8,9]. Spasticity is characterized by a velocity-dependent increase in tonic stretch reflexes (muscle tone) with exaggerated tendon jerks, resulting from hyperexcitability of the stretch reflex, as one component of the upper motor neuron syndrome [10]. Reducing spasticity is therefore one of the main goals of clinical practice in order to enhance posture, mobility, and muscle function, and to reduce pain and improve the quality of life of both patients and caregivers [8,11].

Among the treatments for spasticity, botulinum toxin injection has the strongest level of scientific evidence and has been used for more than 30 years in children with CP, starting from the age of 2 years [12,13].

Botulinum toxin, combined with rehabilitation and other treatments, enhances motor learning, promotes functional progression, and delays orthopedic deformities [14].

Physiologically, acetylcholine (ACh) released at the neuromuscular junction transmits nerve impulses from motor neurons to muscles. Injected in a muscle, the toxin blocks the neuromuscular transmission, causing localized and temporary paralysis. The absence of ACh in the synapse promotes the degradation of nicotinic receptors and blocks neurotransmission. This process is reversible, and muscle function usually returns to baseline within 3–5 months [12].

Target muscles can be identified by palpation, electromyography (EMG), or ultrasound [15]. An anesthetic ointment such as EMLA should be applied at the injection site 60 min before injection [16]. Distraction techniques may also help relieve pain during the session [17].

Infiltration can also occur under sedation in cases of marked psychomotor agitation of the patient and/or in cases of technical difficulties in performing the procedure.

The doses used depend on the severity of the spasticity, the number and size of muscles treated, the weight of the patient, and the type of toxin. For Botox, 3 to 8 units/kg can be used, without exceeding 300 units per session. The dilution is most often 100 units/mL [14].

In the first injection and especially in patients with comorbidities, lower initial doses are recommended. A minimum interval of 3 months is required between two sessions.

The main clinical contraindications are Lambert–Eaton syndrome, amyotrophic lateral sclerosis, myasthenia gravis, anticoagulant use, pregnancy, lactation, and severe respiratory obstruction [18]. Life-threatening complications are not described in the literature, but other complications have been reported: rashes, cramps, flu-like symptoms, pain, or hematomas at the site of injection [14]. Rarely, excessive muscle weakness and asthenia have been reported. There is a risk of falling after triceps are injected, rare urinary leakage or retention in the injection of adductors, and disorders of swallowing in the case of neck muscles [18].

Botulinum toxin injections are routinely administered at our center in pediatric patients with cerebral palsy and spasticity, following international recommendations and standardized protocols for dosage and administration [12,13]. This procedure is part of routine clinical practice at the Pediatric Neurology Unit of Fondazione Policlinico Universitario A. Gemelli. These case series aim to document the presence of transient gastrocnemius muscle fasciculations in the context of spasticity treatment with botulinum toxin and to discuss possible underlying mechanisms and clinical implications.

## 2. Case Presentation

We report three cases of transient gastrocnemius muscle fasciculations, incidentally observed in different patients at separate time points following botulinum toxin injection (with different batches of botulinum toxin). Two of these events occurred in the summer of 2022 and the third event occurred in May 2024 at the Pediatric Neurology Unit of Fondazione Policlinico A. Gemelli, Rome. The first two subjects had never been treated with botulinum toxin before, whereas the third had already undergone two previous injections. Prior to treatment, we conducted a history interview; in the first two cases no recent intake of additional drugs or vaccinations was reported, while in the third case the patient was undergoing somatotropin therapy, which had been discontinued a few days earlier. An EMLA-type anesthetic ointment was placed at the injection site 30–60 min before the injection in the first two cases. In the third case, the procedure was performed under sevoflurane sedation during multiparametric monitoring in the pediatric intensive care unit. We used the same batch of Botox toxin at a dilution of 100 units/2 mL. According to guidelines, the doses were adjusted to the patients’ body weight.

None of the subjects showed signs of dehydration. As part of the clinical assessment, all subjects underwent blood tests after botulinum toxin injection which included tests for electrolytes and CPK levels, the results of which were within the normal range.

All parents provided written informed consent.

### 2.1. Case 1

This is a 15-year-old patient with a past medical history of preterm birth (27 weeks gestational age) and severe neonatal respiratory distress (APGAR score 3–8) who required mechanical ventilation and intensive care. Neuroradiological images reported a pattern of periventricular leukomalacia. He had delayed neurodevelopmental milestones, with independent walking occurring at 2 years of age. The child underwent occupational therapy and physiokinesiotherapy from the first months of life.

At the moment of our assessment (15 years) he reported a pattern of mild spastic diplegia: on passive mobilization, an increased tone was observed in the lower extremities (right > left) resulting in limitation in passive dorsiflexion movement of the tibiotarsal joint (ROM −30 right; −25 left). An initial musculotendinous retraction at the level of the Achilles tendon, bilaterally, was also observed. Autonomous gait was characterized by reduced commuting in the left upper limb and inversion of gait pattern (left > right), with a possible achievement of full plantigrade position in mid-stance. The six-minute walk test (6MWT) showed a total distance of 499 m (average of 84 m/min) with steady gait and in the absence of signs of fatigue. In July 2022 he reported paucisymptomatic SARS-CoV-2 infection with rhinitis, no fever, and no drug intake. In August 2022 he underwent botulinum toxin infiltration after oral premedication with cetirizine 10 mg and betamethasone 1 mg for egg allergy.

He underwent botulinum toxin infiltration proceeding in this order:

1. Right medial gastrocnemius (40 IU)
2. Right lateral gastrocnemius (25 IU)
3. Left medial gastrocnemius (20 IU)

During the second infiltration, fasciculations were observed in the first infiltrated muscle (Video S1). Therefore, we discontinued the infiltration (administered a lower dose—25 IU—than the planned dosage of 40 IU) with muscle stretching of the right calf. Fasciculations at the right medial gastrocnemius persisted for about 20–30 s, with subsequent reduction in the frequency and intensity of muscle contractions until complete resolution. In the meantime, fasciculations of lower intensity were also observed in the second infiltrated muscle site which were resolved after about 10 s. The patient reported no muscle pain, describing a burning sensation at the level of the inoculum site only. Due to the short duration and spontaneous resolution of the episode, infiltration at the level of the third muscle was continued; as fasciculations recurred during inoculation, it was interrupted (administered a lower dose—20 IU—compared to the planned dosage of 40 IU). A therapist initiated muscle stretching of the left calf with complete resolution of symptoms occurring after about 50 s. The patient was monitored for about an hour with no further clinical signs, and no fasciculations were observed in other muscle groups.

At a distance of 24 h, the boy reported no additional contractions or other adverse reactions. After the infiltration, he underwent physiotherapy treatment at a frequency of 4 times/week. Follow-up after two months showed an increase in joint excursion of the ankle joint, better on the right one (ROM −15° right; −21° left). In addition, functional improvement in walking was observed and also reported by the patient. He underwent the six-minute walk test (6MWT): the total meters pursued was 509 (average of 84.8 m/min) with steady gait and in the absence of signs of fatigue. We repeated the injection after six months with a different batch of botulinum toxin, and fasciculation occurred again in the same muscle during the time of inoculation and with the absence of subsequent complications. A similar improvement of motor function as the previous injection was also observed.

### 2.2. Case 2

This is a 13-year-old Russian patient with cri du chat syndrome (carrier of short arm deletion of chromosome 5p14,313.3), diplegia, severe intellectual disability, and visual impairment. His past medical history includes preterm birth (32 weeks gestational age) and severe neonatal respiratory distress. He was adopted at 15 months of age by an Italian family. Due to the presence of motor delay, at the age of 2 years he underwent a brain MRI that showed periventricular leukomalacia due to previous hypoxic-ischemic suffering. He developed spastic diplegia, visual impairment (retinal hypopigmentation and partial optic nerve atrophy), and intellectual disability associated with behavioral features compatible with an autism spectrum disorder. From a genetic point of view, investigations (CGH-array) revealed a duplication of the long arm of chromosome Y11.222 of 804 Kb and a deletion of the short arm of chromosome 5p14.3 of 11.2 Mb which were considered pathogenic and responsible for the clinical signs, consistent with a diagnosis of cri du chat syndrome. He was evaluated in August 2022 at the age of 13 years, presenting an increased muscle tone at the lower limbs (right > left), mainly at the right medial and lateral gastrocnemius muscles and the left medial gastrocnemius muscle with a moderate functional limitation. His parents reported no drug or food allergies. He underwent botulinum toxin infiltration proceeding in this order:

- Medial left gastrocnemius (40 IU);
- Right lateral gastrocnemius (40 IU);
- Right medial gastrocnemius (40 IU).

At the end of the third injection, about 15–20 s later, fasciculations were observed at the injection site, and were more evident on the left side. Bilateral muscle stretching was immediately performed.

The fasciculations persisted for about 6 to 7 min, with a progressive reduction in terms of frequency and intensity, until complete resolution. They were not associated with other clinical signs, and no fasciculations were observed in other muscle groups.

The boy was monitored for about one hour, with no further adverse reactions, no pain, and no functional deficits. At a distance of 24 h, the boy reported no additional involuntary contractions or other adverse reactions. After the infiltration, the patient intensified physiotherapy treatment at a frequency of 4 times/week. At follow-up (two months later), an improvement of ankle joint excursion was observed, with a possibility of reaching neutral position (90°), bilaterally (left > right), and a reduction in muscle spasticity at the gastrocnemius muscles, bilaterally. Functional gait improvement was also observed, with better orientation of the right foot and reduction in varus-supination stance. Behavioral difficulties and visual impairment of the patient did not allow any further structured assessment. We repeated the injection after six months with a different batch of botulinum toxin, and fasciculation occurred again in the same muscle during the time of inoculation and in the absence of subsequent complications.

### 2.3. Case 3

This is an 8-year-old patient born in Brazil who moved to Italy with her family at the age of 3 years with a past medical history of intrauterine growth restriction and prematurity (28 weeks gestational age). Her birth weight was 620 g. She was admitted to the Neonatal Intensive Care Unit for one month due to bronchopulmonary dysplasia and retinopathy of prematurity. She has undergone endocrinological follow-up since 2021 for growth hormone deficiency in replacement treatment with Somatotropin and early puberty. For suspected Silver–Russell syndrome, she underwent array-CGH and exome with negative results. Her parents reported normal psychomotor development in the first months. In December 2019, she was evaluated for the first time at our clinic due to alterations in stability and balance with evidence of a postural-motor pattern of mild diplegia with greater involvement of the left lower limb. The brain MRI performed at 2 years documented sporadic and blurred areolae of hyperintensity in the bilateral posterior white matter (right > left) associated with mild ectasia of the ventricular trigones suggestive of previous mild tissue damage (probably perinatal).

Since 2021 she has undergone a rehabilitation treatment of physiotherapy and neuropsychomotor and speech therapy. In May 2023, due to a worsening of the walking pattern and progression of the muscular tension affecting the left medial gastrocnemius muscle, she underwent the first botulinum toxin infiltration (Botox 100 IU in 2 cc of SF) affecting the left medial (30 IU) and left lateral (25 IU) gastrocnemius muscles in the absence of periprocedural complications and with partial clinical benefit.

In November 2023 the girl underwent a second infiltration of botulinum toxin (Botox 100 IU in 2 cc of SF) under sedation with sevoflurane of left lateral gastrocnemius muscle (30 IU), left medial gastrocnemius muscle (30 IU) and right medial gastrocnemius muscle (30 IU) without periprocedural complications and a stability of postural-motor picture of mild diplegia with greater involvement of the left lower limb.

The third botulinum toxin infiltration (Botox 100 IU in 2 cc of SF) was performed in May 2024 under sedation with sevoflurane proceeding in this order:

- Left lateral gastrocnemius muscle (30 IU),
- Left medial gastrocnemius muscle (30 IU),
- Right medial gastrocnemius muscle (30 IU).

Following the infiltration of botulinum toxin, an episode of fasciculations was observed in the belly of the right medial gastrocnemius muscle which decreased in frequency and intensity and resolved in approximately 2–3 min following stretching of the district (Video S2). No fasciculations were observed in other muscle groups. No adverse reactions were observed either from the anesthesia or from the procedure performed. She reported tenderness and a burning sensation at the injection site in both medial gastrocnemius muscles, which decreased with mobilization.

We monitored the patient for about an hour, observing no further adverse reactions. The patient reported no additional complications at a distance of 24 h. Her parents reported no allergies to drugs and/or foods or infections in the previous months. No blood tests including tests for electrolytes were performed. After the infiltration, she attended physiotherapy four times per week.

At follow-up one month later, we observed an improvement of the left ankle joint excursion. Due to behavioral difficulties, no structured motor functional assessments could be performed.

## 3. Discussion

In these three pediatric cases, we observed transitory muscle fasciculations at the sites of inoculation of botulinum toxin injection that we considered as a possible botulinum-related side effect. For this reason, the Pharmacovigilant Unit of our hospital was informed and an alert was sent to the Italian medicines agency (AIFA).

Fasciculation describes the involuntary and irregular activation of a single motor unit. During muscular activity it is important that the neuronal membrane maintains its resting potential. This is achieved through the constant efflux of Na+ ions and influx of K+ ions in a ratio of 3:2 via the ATPase pump, thereby reversing the flow of cations that occur during the course of an action potential [19]. This mechanism, in turn, depends on the action of acetylcholine on the receptors at the neuromuscular junction. When this mechanism does not work, neuronal hyperexcitability and the appearance of involuntary contractions of individual muscles (fasciculation) can occur. This condition is observed in several neurological conditions such as amyotrophic lateral sclerosis (ALS), spinobulbar muscular atrophy, peripheral neuropathies, and multifocal motor neuropathy. Fasciculations are also observed in conditions such as hyperthyroidism, anxiety, electrolyte imbalances, neurotransmitter dysfunctions (ACh), excessive physical exertion, hypoglycemia, potassium deficiency, and hypomagnesemia. In some cases, they may reflect an undesirable effect of medications (albuterol, ACh Esterase Inhibitors, or benzodiazepines) and stimulating substances (caffeine, amphetamines, and during drug or alcohol withdrawal).

In the three reported cases, a healthcare professional reported unexpected, transitory, and non-harmful fasciculations after the botulinum toxin infiltration performed at the planned dosages, requiring extended monitoring and stretching. No further significant clinical conditions were observed, including changes in muscle tone, strength, or functional outcomes. In all the three patients, the fasciculation occurred during the reported injections only, with spontaneous resolution. In the three reported cases, the amounts of botulinum toxin infiltrated were appropriate for the patients’ weights, thus excluding a condition due to an “overdose” of the toxin. However, a possible influence of the botulinum toxin dosage on the occurrence of fasciculations cannot be ruled out.

In the first case, a SARS-CoV-2 infection was reported three weeks before the botulinum injection, therefore a possible neurological manifestation of the infection itself was considered. However, no similar cases are described in the literature, and furthermore, until the time of inoculation, no neuromuscular symptoms had been reported. Following the second episode, we viewed recent electrolyte tests that were normal.

These events are consistent with mild adverse events possibly related to botulinum toxin infiltration. Subsequent assessments confirmed their transient and non-harmful nature, as well as the expected benefits for posture, mobility, and muscle function in all three patients. The first two patients were also re-evaluated at 12 months, with no adverse events reported.

A single case has been described in India in a subject with CP who manifested generalized fasciculations as a rare manifestation of foodborne botulism [20]. This was a case of a 35-year-old male who presented with sudden-onset abdominal pain following the consumption of stale food. The symptoms were vomiting, bilateral symmetrical drooping of eyelids with blurring of vision, and difficulty in swallowing. He developed weakness of both upper limbs followed by lower limbs. Motor system examination showed normal tone with bilateral symmetrical quadriparesis and generalized fasciculations involving the anterior chest wall and bilateral upper and lower limbs. The biochemical parameters and electrolyte levels were not altered, but the patient had an elevated serum CPK level (793 IU/L). He was treated with intravenous hydrocortisone and antibiotics. Further clinical causes were ruled out. In the literature, there have been very few cases of botulinum toxin food poisoning with a clinical presentation predominantly featuring fasciculations [21].

In addition, no cases of fasciculations have been found in the literature as a side effect of intramuscular injection in children with CP; therefore, the occurrence of fasciculations cannot be attributed to the infiltration technique.

A possible hypothesis could be related to a change in local neuromuscular transmission due to the effect of botulinum toxin influencing the motor nerves transmitting signals to muscles at the neuromuscular junction by altering the release of Ach. This could induce subclinical neuromuscular transmission abnormalities in non-injected regions; in fact, in rare cases botulinum toxin may spread beyond the injection site through passive diffusion, hematogenous dissemination, or retrograde axonal transport [22,23]. Furthermore, it has been reported that repeated cycles of chemodenervation and subsequent reinnervation may induce chronic alterations in motor endplate morphology, Ach-receptor density, and overall neuromuscular junction stability [22,23,24]. In a recent study on the use of botulinum toxin in facial spasms, spontaneous muscle activity, such as myokymia and fasciculations, was observed in some patients undergoing repeated botulinum toxin treatment. Although clinically subtle, these symptoms may reflect possible synaptic reorganization, axonal sprouting, or heightened cholinergic-receptor sensitivity [24]. However, in the present case reports, the children received the botulinum toxin for the first time except for case 3 (where the child received it for the third time); therefore, this effect due to repeated infiltration could not be completely applicable in our cases.

The present case reports have limitations that should be acknowledged. First, it describes three pediatric cases only and it does not allow for the broad generalization of the findings to the overall use of botulinum toxin. Second, laboratory and instrumental assessments were limited; in particular, no neurophysiological recordings were obtained during the episodes, which would have enabled a more precise characterization of the events. Despite these constraints, the case series provides novel clinical observations that may inform future systematic investigations of this potential adverse effect. At present, it is not possible to establish correlations with patient age, the severity of cerebral palsy, or the specific muscles injected. This side-effect of botulinum toxin injection would appear to be a transient event without impact on clinical outcomes. Therefore, pediatric injection protocols would not require modification, apart from informing parents before the procedure to reassure them about the possible transient and non-harmful nature of this event.

## 4. Conclusions

Muscle fasciculation can be benign or be associated with specific neurological disorders, including spinal muscular atrophy, juvenile ALS, Charcot–Marie–Tooth disease, central nervous system lesions, and a wide range of genetic and metabolic conditions [25].

A systematic review of the studies available to date shows limited evidence of a fasciculations as a direct side-effect of botulinum toxin infusion [20,21,22,23,24].

The pathophysiological mechanism, leading to the onset of muscle fasciculations following a blockage of acetylcholine release, is unclear. The combined observation of subclinical transmission abnormalities and spontaneous muscle activity on the non-injected side could provide compelling evidence of regional toxin dissemination [22,23,24].

In specific patients, botulinum toxin administration may induce measurable neuromuscular changes. These results underscore the importance of individualized dose titration and careful injection planning.

However, the common clinical features of the three cases and their follow-up over time allowed firstly the confirmation of the transient and non-harmful nature of the adverse event and secondly the confirmation of the expected benefits which support the use of botulinum toxin infiltration, combined with rehabilitation, as a choice treatment for spasticity. Further studies are needed to clarify the causal relationship between the onset of fasciculations and botulinum toxin injection.

## Data Availability

The original contributions presented in this study are included in the article/Supplementary Material. Further inquiries can be directed to the corresponding authors.

## Supplementary Materials

The following supporting information can be downloaded at https://www.mdpi.com/article/10.3390/brainsci15101048/s1: Video S1: Case 1; Video S2: Case 3.

## Abbreviations

The following abbreviations are used in this manuscript:

CP	Cerebral palsy
ACh	Acetylcholine
EMG	Electromyography
EMLA	Lidocaine anesthetic cream
6MWT	Six-minute walk test
IU	International Units
ROM	Range of motion
MRI	Magnetic Resonance Imaging
ROP	Retinopathy of prematurity
FOP	Patent foramen ovale
PEG	Percutaneous gastrostomy
SF	Physiological Solution
Na+	Sodium
K+	Potassium
ALS	Amyotrophic lateral sclerosis
CPK	Creatine Phosphokinase
